# Tsallis Entropy for Loss Models and Survival Models Involving Truncated and Censored Random Variables

**DOI:** 10.3390/e24111654

**Published:** 2022-11-14

**Authors:** Vasile Preda, Silvia Dedu, Iuliana Iatan, Ioana Dănilă Cernat, Muhammad Sheraz

**Affiliations:** 1 “Gheorghe Mihoc-Caius Iacob” Institute of Mathematical Statistics and Applied Mathematics, 050711 Bucharest, Romania; 2 “Costin C. Kiriţescu” National Institute of Economic Research, 050711 Bucharest, Romania; 3 Faculty of Mathematics and Computer Science, University of Bucharest, Academiei 14, 010014 Bucharest, Romania; 4 Department of Applied Mathematics, Bucharest University of Economic Studies, 010734 Bucharest, Romania; 5 Department of Mathematics and Computer Science, Technical University of Civil Engineering, 020396 Bucharest, Romania; 6 Institute of Business Administration Karachi, Department of Mathematical Sciences, School of Mathematics and Computer Science, Karachi 75270, Pakistan; 7 Department Financial Mathematics, Fraunhofer ITWM, Fraunhofer-Platz, 67663 Kaiserslautern, Germany

**Keywords:** Tsallis entropy measures, risk assessment, survival models, loss models, truncation, censoring, proportional hazard model, proportional reversed hazard model

## Abstract

The aim of this paper consists in developing an entropy-based approach to risk assessment for actuarial models involving truncated and censored random variables by using the Tsallis entropy measure. The effect of some partial insurance models, such as inflation, truncation and censoring from above and truncation and censoring from below upon the entropy of losses is investigated in this framework. Analytic expressions for the per-payment and per-loss entropies are obtained, and the relationship between these entropies are studied. The Tsallis entropy of losses of the right-truncated loss random variable corresponding to the per-loss risk model with a deductible *d* and a policy limit *u* is computed for the exponential, Weibull, $\chi^{2}$ or Gamma distribution. In this context, the properties of the resulting entropies, such as the residual loss entropy and the past loss entropy, are studied as a result of using a deductible and a policy limit, respectively. Relationships between these entropy measures are derived, and the combined effect of a deductible and a policy limit is also analyzed. By investigating residual and past entropies for survival models, the entropies of losses corresponding to the proportional hazard and proportional reversed hazard models are derived. The Tsallis entropy approach for actuarial models involving truncated and censored random variables is new and more realistic, since it allows a greater degree of flexibility and improves the modeling accuracy.

## 1. Introduction

Risk assessment represents an important topic in various fields, since it allows designing the optimal strategy in many real-world problems. The fundamental concept of entropy can be used to evaluate the uncertainty degree corresponding to the result of an experiment, phenomenon or random variable. Recent research results in statistics prove the increased interest for using different entropy measures. Many authors have dealt with this matter, among them are Koukoumis and Karagrigoriou [1], Iatan et al. [2], Li et al. [3], Miśkiewicz [4], Toma et. al. [5], Moretto et al. [6], Remuzgo et al. [7], Sheraz et al. [8] and Toma and Leoni-Aubin [9]. One of the most important information measures, the Tsallis entropy, has attracted considerable interest in statistical physics and many other fields as well. We can mention here the contributions of Nayak et al. [10], Pavlos et al. [11] and Singh and Cui [12]. Recently, Balakrishnan et al. [13] proposed a general formulation of a class of entropy measures depending on two parameters, which includes Shannon, Tsallis and fractional entropy as special cases.

As entropy can be regarded as a measure of variability for absolutely continuous random variables or measure of variation or diversity of the possible values of a discrete random variable, it can be used for risk assessment in various domains. In actuarial science, one of the main objectives which defines the optimal strategy of an insurance company is directed towards minimizing the risk of the claims. Ebrahimi [14] and Ebrahimi and Pellerey [15] studied the problem of measuring uncertainty in life distributions. The uncertainty corresponding to loss random variables in actuarial models can be evaluated also by the entropy of the loss distribution. Frequently in actuarial practice, as a consequence of using deductibles and policy limits, the practitioners have to deal with transformed data, generated by truncation and censoring. Baxter [16] and Zografos [17] developed information measure methods for mixed and censored random variables, respectively. The entropic approach enables the assessment of the uncertainty degree for loss models involving truncated and censored random variables. Sachlas and Papaioannou [18] investigated the effect of inflation, truncation or censoring from below or above on the Shannon entropy of losses of insurance policies. In this context of per-payment and per-loss models, they derived analytic formulas for the Shannon entropy of actuarial models involving several types of partial insurance coverage and studied the properties of the resulting entropies. Recent results in this field have also been obtained by Gupta and Gupta [19] and Di Crescenzo and Longobardi [20], Meselidis and Karagrigoriou [21].

This paper aims to develop several entropy-based risk models involving truncated and censored loss random variables. In this framework, the effect of some partial insurance schemes, such truncation and censoring from above, truncation and censoring from below and inflation is investigated using the Tsallis entropy. The paper is organized as follows. In Section 2 some preliminary results are presented. In Section 3 representation formulas for the Tsallis entropy corresponding to the truncated and censored loss random variables in the per-payment and per-loss approach are derived, and the relationships between these entropies are obtained. Moreover, the combined effect of a deductible and a policy limit is investigated. In Section 4, closed formulas for the Tsallis entropy corresponding to some survival models are derived, including the proportional hazard and the proportional reversed hazard models. Some concluding remarks are provided in the last section.

## 2. Preliminaries

### 2.1. The Exponential Distribution

An exponential distributed random variable X∼Exp(λ) is defined by the probability density function:

(1) $$f\left(x\right)=\left\{\begin{array}{c} \lambda \cdot e^{-\lambda x},\;if\;\;x\geq 0\\ 0,\;\;if\;\;x<0, \end{array}\right.$$

with λ∈R,λ>0 and the cumulative distribution function:

(2) $$F_{X}\left(x\right)=1-e^{-\lambda x},\;x\geq 0.$$

### 2.2. The Weibull Distribution

A Weibull distributed random variable X∼W(α,λ,γ) is closely related to an exponential distributed random variable and has the probability density function:

(3) $$f\left(x\right)=\left\{\begin{array}{c} \gamma \lambda {\left(x-\alpha \right)}^{\gamma -1}\cdot e^{-\lambda {\left(x-\alpha \right)}^{\gamma }},\;if\;\;x\geq \alpha \\ 0,\;\;if\;\;x<\alpha , \end{array}\right.$$

with α,λ,γ∈R,λ,γ > 0.

If X∼Exp(1), then the Weibull distribution can be generated using the formula:

(4) $$W=\alpha +{\left(\frac{X}{\lambda }\right)}^{\frac{1}{\gamma }}.$$

### 2.3. The $\chi^{2}$ Distribution

Let $Z_{i},\;1\leq i\leq \gamma$ be independent random variables, Gaussian distributed and N(0,1). A random variable $\chi^{2}$ with γ degrees of freedom can be represented as:

(5) $$\chi^{2}=\sum_{i=1}^{\gamma }Z_{i}^{2},\;\gamma \in N^{*}.$$

A $\chi^{2}$ distributed random variable with γ degrees of freedom is represented by the probability density function:

(6) $$f\left(x\right)=\frac{1}{2^{\frac{\gamma }{2}}\Gamma \left(\frac{\gamma }{2}\right)}x^{\frac{\gamma }{2}-1}e^{-\frac{x}{2}},\;x\geq 0,$$

where Γ denotes the Euler Gamma function.

### 2.4. The Gamma Distribution

An exponential distributed random variable X∼G(α,λ,γ) is defined by the probability density function [22]:

(7) $$f\left(x\right)=\left\{\begin{array}{c} \frac{\lambda^{\gamma }}{\Gamma (\gamma )}{\left(x-\alpha \right)}^{\gamma -1}\cdot e^{-\lambda (x-\alpha )},\;if\;\;x\geq \alpha \\ 0,\;\;if\;\;x<\alpha , \end{array}\right.$$

where α∈R,γ,λ>0 are, respectively, the location parameter, the scale parameter and the form parameter of the variable *X*.

We can notice that an exponential distributed random variable is a gamma random variable G(0,λ,1) and a $\chi^{2}$ distributed random variable is a gamma distributed random variable $G(0,\;\frac{1}{2},\;\frac{\gamma }{2})$.

If $Y∼G(\alpha ,\;\lambda ,\;\frac{\gamma }{2})$ and $Z∼G(0,\;\frac{1}{2},\;\frac{\gamma }{2})$, then we have:

(8) $$Y=\alpha +\frac{Z}{2\lambda }.$$

### 2.5. The Tsallis Entropy

Entropy represents a fundamental concept which can be used to evaluate the uncertainty associated with a random variable or with the result of an experiment. It provides information regarding the predictability of the results of a random variable *X*. The Shannon entropy, along with other measures of information, such as the Renyi entropy, may be interpreted as a descriptive quantity of the corresponding probability density function.

Entropy can be regarded as a measure of variability for absolutely continuous random variables or as a measure of variation or diversity of the possible values of discrete random variables. Due to the widespread applicability and use of information measures, the derivation of explicit expressions for various entropy and divergence measures corresponding to univariate and multivariate distributions has been a subject of interest; see, for example, Pardo [23], Toma [24], Belzunce et al. [25], Vonta and Karagrigoriou [26]. Various measures of entropy and generalizations thereof have been proposed in the literature.

The Tsallis entropy was introduced by Constantino Tsallis in 1988 [27,28,29,30] with the aim of generalizing the standard Boltzmann–Gibbs entropy and, since then, it has attracted considerable interest in the physics community, as well as outside it. Recently, Furuichi [31,32] investigated information theoretical properties of the Tsallis entropy and obtained a uniqueness theorem for the Tsallis entropy. The use of Tsallis entropy enhances the analysis and solving of some important problems regarding financial data and phenomena modeling, such as the distribution of asset returns, derivative pricing or risk aversion. Recent research in statistics increased the interest in using Tsallis entropy. Trivellato [33,34] used the minimization of the divergence corresponding to the Tsallis entropy as a criterion to select a pricing measure in the valuation problems of incomplete markets and gave conditions on the existence and on the equivalence to the basic measure of the minimal *k*–entropy martingale measure. Preda et al. [35,36] used Tsallis and Kaniadakis entropies to construct the minimal entropy martingale for semi-Markov regime switching interest rate models and to derive new Lorenz curves for modeling income distribution. Miranskyy et al. [37] investigated the application of some extended entropies, such as Landsberg–Vedral, Rényi and Tsallis entropies to the classification of traces related to various software defects.

Let *X* be a real-valued discrete random variable defined on the probability space (Ω,F,P), with the probability mass function $p_{X}$. Let α∈R∖{1}. We introduce the definition of Tsallis entropy [27] for discrete and absolutely continuous random variables in terms of expected value operator with respect to a probability measure.

**Definition 1.**  *The Tsallis entropy corresponding to the discrete random variable X is defined by:*

(9) $$H_{\alpha }^{T}\left(X\right)=-E_{p_{X}}\left[\frac{p_{X}{\left(x\right)}^{\alpha -1}-1}{\alpha -1}\right],$$

*where $E_{p_{X}}\left[\cdot \right]$ represents the expected value operator with respect to the probability mass function $p_{X}$.*

Let *X* be a real-valued continuous random variable defined on the probability space (Ω,F,P), with the probability density function $f_{X}$. Let α∈R∖{1}.

**Definition 2.**  *The Tsallis entropy corresponding to the continuous random variable X is defined by:*

(10) $$H_{\alpha }^{T}\left(f_{X}\right)=-E_{f_{X}}\left[\frac{f_{X}{\left(x\right)}^{\alpha -1}-1}{\alpha -1}\right],$$

*provided that the integral exists, where $E_{f_{X}}\left[\cdot \right]$ represents the expected value operator with respect to the probability density function $f_{X}$.*

In the sequel, we suppose to know the properties of the expected value operator, such as additivity and homogeneity.

Note that for α=2, the Tsallis entropy reduces to the second-order entropy [38] and for α→1, we obtain the Shannon entropy [39]. The real parameter α was introduced in the definition of Tsallis entropy for evaluating more accurately the degree of uncertainty. In this regard, the Tsallis parameter tunes the importance assigned to rare events in the considered model.

Highly uncertain insurance policies are less reliable. The uncertainty for the loss associated to an insurance policy can be quantified by using the entropy of the corresponding loss distribution. In the actuarial practice, frequently transformed data are available as a consequence of deductibles and liability limits. Recent research in statistics increased the interest for using different entropy measures for risk assessment.

## 3. Tsallis Entropy Approach for Loss Models

We denote by *X* the random variable which models the loss corresponding to an insurance policy. We suppose that *X* is non-negative and denote by $f_{X}$ and $F_{X}$ its probability density function and cumulative distribution function, respectively. Let $S_{X}$ be the survival function of the random variable *X*, defined by $S_{X}\left(x\right)=P\left(X>x\right)$.

We consider truncated and censored random variables obtained from *X*, which can be used to model situations which frequently appear in actuarial practice as a consequence of using deductibles and policy limits. In the next subsections, analytical expressions for the Tsallis entropy are derived, corresponding to the loss models based on truncated and censored random variables.

### 3.1. Loss Models Involving Truncation or Censoring from Below

Loss models with left-truncated or censored from below random variables are used when losses are not recorded or reported below a specified threshold, mainly as a result of applying deductible policies. We denote by *d* the value of the threshold, referred to as the deductible value. According to Kluggman et al. [40], there are two approaches used to express the random variable which models the loss, corresponding to the per-payment and per-loss cases, respectively.

In the per-payment case, losses or claims below the value of the deductible may not be reported to the insurance company, generating truncated from below or left-truncated data.

We denote by $X_{lt}\left(d\right)$ the left-truncated random variable which models the loss corresponding to an insurance policy with a deductible *d* in the per-payment case. It can be expressed as $X_{lt}\left(d\right)=\left[X|X>d\right]$, or equivalently:

(11) $$X_{lt}\left(d\right)=\left\{\begin{array}{c} X,\;if\;\;X>d\\ \mathrm{not}\;\mathrm{defined},\;\;if\;\;X\leq d. \end{array}\right.$$

In order to investigate the effect of truncation from bellow, we use the Tsallis entropy for evaluating uncertainty corresponding to the loss covered by the insurance company. The following theorem establishes the relationship between the Tsallis entropy of the random variables *X* and $X_{lt}\left(d\right)$. We denote by $H_{\alpha }^{T}\left(X_{lt}\left(d\right)\right)$ the per-payment Tsallis entropy with a deductible *d*.

We denote by $I_{A}$ the indicator function of the set *A*, defined by:

(12) $$I_{A}\left(x\right)=\left\{\begin{array}{c} 1,\;if\;\;x\in A\\ 0,\;\;otherwise. \end{array}\right.$$

In the sequel, the integrals are always supposed to be correctly defined.

**Theorem 1.**  *Let X be a non-negative random variable which models the loss corresponding to an insurance policy. Let α∈R∖{1} and d>0. The Tsallis entropy $H_{\alpha }^{T}\left(X_{lt}\left(d\right)\right)$ of the left-truncated loss random variable corresponding to the per-payment risk model with a deductible d can be expressed as follows:*

(13) $$H_{\alpha }^{T}\left(X_{lt}\left(d\right)\right)=S_{X}^{-\alpha }\left(d\right)\left(H_{\alpha }^{T}\left(X\right)+E_{f_{X}}\left[\frac{f_{X}^{\alpha -1}\left(x\right)-1}{\alpha -1}I_{\left\{0<X<d\right\}}\right]\right)+\frac{1-S_{X}^{1-\alpha }\left(d\right)}{\alpha -1}.$$

**Proof.**  The probability density function of the random variable $X_{lt}\left(d\right)$ is given by $f_{X_{lt}\left(d\right)}\left(x\right)=\frac{f_{X}\left(x\right)}{S_{X}\left(d\right)}$, x>d. Therefore, the Tsallis entropy of the random variable $X_{lt}\left(d\right)$ can be expressed as follows:

$$H_{\alpha }^{T}\left(X_{lt}\left(d\right)\right)=-\frac{1}{\left(\alpha -1\right)S_{X}\left(d\right)}E_{f_{X}}\left[\left({\left(\frac{f_{X}\left(x\right)}{S_{X}\left(d\right)}\right)}^{\alpha -1}-1\right)I_{\left\{d<X<\infty \right\}}\right]$$

$$=-\frac{1}{S_{X}\left(d\right)}\left(S_{X}^{{}^{1-\alpha }}\left(d\right)E_{f_{X}}\left[\frac{f_{X}^{\alpha -1}\left(x\right)-1}{\alpha -1}I_{\left\{d<X<\infty \right\}}\right]+\frac{S_{X}^{1-\alpha }\left(d\right)-1}{\alpha -1}E_{f_{X}}\left[I_{\left\{d<X<\infty \right\}}\right]\right)$$

$$=-\frac{1}{S_{X}\left(d\right)}\left(S_{X}^{{}^{1-\alpha }}\left(d\right)E_{f_{X}}\left[\frac{f_{X}^{\alpha -1}\left(x\right)-1}{\alpha -1}\right]-S_{X}^{{}^{1-\alpha }}\left(d\right)E_{f_{X}}\left[\frac{f_{X}^{\alpha -1}\left(x\right)-1}{\alpha -1}I_{\left\{0<X<d\right\}}\right]\right)-$$

$$-\frac{1}{S_{X}\left(d\right)}\left(\frac{S_{X}^{1-\alpha }\left(d\right)-1}{\alpha -1}E_{f_{X}}\left[I_{\left\{d<X<\infty \right\}}\right]\right)=$$

$$=S^{{}^{-\alpha }}\left(d\right)\left(H_{\alpha }^{T}\left(X\right)+E_{f_{X}}\left[\frac{f_{X}^{\alpha -1}\left(x\right)-1}{\alpha -1}I_{\left\{0<X<d\right\}}\right]\right)+\frac{1-S_{X}^{1-\alpha }\left(d\right)}{\alpha -1}.$$

□

**Remark 1.**  *For the limiting case α→1, we obtain the corresponding results for the Shannon entropy from* [18].

In the per-loss case corresponding to an insurance policy with a deductible *d*, all the claims are reported, but only the ones over the deductible value are paid. As only the real losses of the insurer are taken into consideration, this situation generates censored from below data.

We denote by $X_{lc}\left(d\right)$ the left-censored random variable which models the loss corresponding to an insurance policy with a deductible *d* in the per-loss case. As *X* is censored from below at point *d*, it results that the random variable $X_{lc}\left(d\right)$ can be expressed as follows:

(14) $$X_{lc}\left(d\right)=\left\{\begin{array}{c} X,\;if\;\;X>d\\ 0,\;\;if\;\;X\leq d. \end{array}\right.$$

We note that $X_{lc}\left(d\right)$ assigns a positive probability mass at zero point, corresponding to the case X≤d. In this case, $X_{lc}\left(d\right)$ it not absolutely continuous, but a mixed random variable, consisting of a discrete and a continuous part. We can remark that the per-payment loss random variable can be expressed as the per-loss one given that the later is positive.

In the next theorem, the relation between the Tsallis entropy of the random variables *X* and $X_{lc}\left(d\right)$ is established.

**Theorem 2.**  *Let X be a non-negative random variable which models the loss corresponding to an insurance policy. Let α∈R∖{1} and d>0. The Tsallis entropy $H_{\alpha }^{T}\left(X_{lc}\left(d\right)\right)$ of the left-censored loss random variable corresponding to the per-payment risk model with a deductible d can be expressed as follows:*

(15) $$H_{\alpha }^{T}\left(X_{lc}\left(d\right)\right)=H_{\alpha }^{T}\left(X\right)+E_{f_{X}}\left[\frac{f_{X}^{\alpha -1}\left(x\right)-1}{\alpha -1}I_{\left\{0<X<d\right\}}\right]-F_{X}\left(d\right)\frac{F_{X}^{\alpha -1}\left(d\right)-1}{\alpha -1}.$$

**Proof.**  The Tsallis entropy of $X_{lc}\left(d\right)$, which is a mixed random variable consisting of a discrete part at zero and a continuous part over (d,+∞), is given by:

$$H_{\alpha }^{T}\left(X_{lc}\left(d\right)\right)=-E_{f_{X}}\left[\frac{f_{X}^{\alpha -1}\left(x\right)-1}{\alpha -1}I_{\left\{d<X<\infty \right\}}\right]-F_{X}\left(d\right)\frac{F_{X}^{\alpha -1}\left(d\right)-1}{\alpha -1}$$

$$=-E_{f_{X}}\left[\frac{f_{X}^{\alpha -1}\left(x\right)-1}{\alpha -1}\right]+E_{f_{X}}\left[\frac{f_{X}^{\alpha -1}\left(x\right)-1}{\alpha -1}I_{\left\{0<X<d\right\}}\right]-F_{X}\left(d\right)\frac{F_{X}^{\alpha -1}\left(d\right)-1}{\alpha -1}$$

and the conclusion follows. □

**Remark 2.**  *Let α∈R∖{1} and d>0. Then,*

$$H_{\alpha }^{T}\left(X_{lc}\left(d\right)\right)-H_{\alpha }^{T}\left(X\right)=E_{f_{X}}\left[\frac{f_{X}^{\alpha -1}\left(x\right)-1}{\alpha -1}I_{\left\{0<X<d\right\}}\right]-F_{X}\left(d\right)\frac{F_{X}^{\alpha -1}\left(d\right)-1}{\alpha -1}.$$

*It results that the Tsallis entropy of the left-censored loss random variable corresponding to the per-loss risk model is greater than the Tsallis entropy of the loss random variable, and the difference can be quantified by the right-hand side of the formula above.*

Let λ>0, α∈R∖{1} and d>0. Let *X* be Exp(λ) distributed and denoted by $\varphi_{lc}\left(d,\alpha ,\lambda \right)=H_{\alpha }^{T}\left(X_{lc}\left(d\right)\right)-H_{\alpha }^{T}\left(X\right)$. Using Theorem 2, we obtain

$$\varphi_{lc}\left(d,\alpha ,\lambda \right)=\frac{1}{\alpha -1}\left[\frac{1}{\alpha \lambda }\left(\alpha \lambda e^{-d\lambda }-\lambda^{\alpha }e^{-d\alpha \lambda }+\lambda^{\alpha }-\alpha \lambda \right)-e^{-d\lambda }-{\left(1-e^{-d\lambda }\right)}^{\alpha }+1\right].$$

**Figure 1:**
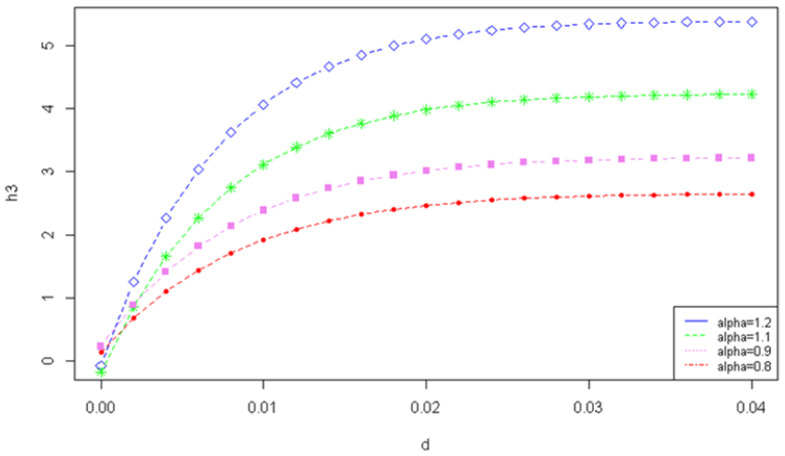
The graph for λ=100 and different values of the Tsallis entropy parameter α.

Figure 1 displays the graph of $\varphi_{lc}$ function for λ=100 and different values of the Tsallis entropy parameter α.

**Theorem 3.**  *Let X be a non-negative random variable which models the loss corresponding to an insurance policy. Let α∈R∖{1}. The Tsallis entropy measures $H_{\alpha }^{T}\left(X_{lt}\left(d\right)\right)$ and $H_{\alpha }^{T}\left(X_{lc}\left(d\right)\right)$ are connected through the following relationship:*

(16) $$S^{\alpha }\left(d\right)\cdot H_{\alpha }^{T}\left(X_{lt}\left(d\right)\right)-H_{\alpha }^{T}\left(X_{lc}\left(d\right)\right)=-H_{\alpha }^{T}\left(B\left(F(d)\right)\right),$$

*where $B\left(F_{X}\left(d\right)\right)$ represents a Bernoulli distributed random variable with parameter $F_{X}\left(d\right)$.*

**Proof.**  By multiplying (13) with $S^{\alpha }\left(d\right)$, we obtain:

$$S_{X}^{\alpha }\left(d\right)\cdot H_{\alpha }^{T}\left(X_{lt}\left(d\right)\right)=H_{\alpha }^{T}\left(X\right)+E_{f_{X}}\left[\frac{f_{X}^{\alpha -1}\left(x\right)-1}{\alpha -1}I_{\left\{0<X<d\right\}}\right]-S_{X}^{\alpha }\left(d\right)\frac{S_{X}^{\alpha -1}\left(d\right)-1}{\alpha -1}.$$

From Theorem 2, we have:

$$H_{\alpha }^{T}\left(X_{lc}\left(d\right)\right)=H_{\alpha }^{T}\left(X\right)+E_{f_{X}}\left[\frac{f_{X}^{\alpha -1}\left(x\right)-1}{\alpha -1}I_{\left\{0<X<d\right\}}\right]-F_{X}\left(d\right)\frac{F_{X}^{\alpha -1}\left(d\right)-1}{\alpha -1}.$$

By subtracting the two relations above, we obtain:

$$S_{X}^{\alpha }\left(d\right)\cdot H_{\alpha }^{T}\left(X_{lt}\left(d\right)\right)-H_{\alpha }^{T}\left(X_{lc}\left(d\right)\right)=S_{X}\left(d\right)\frac{S_{X}^{\alpha -1}\left(d\right)-1}{\alpha -1}+$$

$$+F_{X}\left(d\right)\frac{F_{X}^{\alpha -1}\left(d\right)-1}{\alpha -1}=-H_{\alpha }^{T}\left(B\left(F_{X}\left(d\right)\right)\right).$$

□

Now, we denote by $\lambda \left(x\right)=\frac{f_{X}\left(x\right)}{S_{X}\left(x\right)}$, for $S_{X}\left(x\right)>0$, the hazard rate function of the random variable *X*. In the next theorem, the per-payment simple or residual entropy with a deductible *d* is expressed in terms of the hazard or risk function of *X*.

**Theorem 4.**  *Let X be a non-negative random variable which models the loss corresponding to an insurance policy. Let α∈R∖{1}. The Tsallis entropy of the left-truncated loss random variable corresponding to the per-payment risk model with a deductible d is given by:*

(17) $$H_{\alpha }^{T}\left(X_{lt}\left(d\right)\right)=-S_{X}^{-\alpha }\left(d\right)E_{f_{X}}\left[\frac{\lambda^{\alpha -1}\left(x\right)-1}{\alpha -1}S_{X}^{\alpha -1}\left(x\right)I_{\left\{d<X<\infty \right\}}\right]+\frac{1}{\alpha }.$$

**Proof.**  From Theorem 1, we have:

$$H_{\alpha }^{T}\left(X_{lt}\left(d\right)\right)=S_{X}^{-\alpha }\left(d\right)\left(H_{\alpha }^{T}\left(X\right)+E_{f_{X}}\left[\frac{f_{X}^{\alpha -1}\left(x\right)-1}{\alpha -1}I_{\left\{0<X<d\right\}}\right]\right)+\frac{1-S_{X}^{1-\alpha }\left(d\right)}{\alpha -1}$$

$$=S_{X}^{-\alpha }\left(d\right)\left(-E_{f_{X}}\left[\frac{f_{X}^{\alpha -1}\left(x\right)-1}{\alpha -1}\right]+E_{f_{X}}\left[\frac{f_{X}^{\alpha -1}\left(x\right)-1}{\alpha -1}I_{\left\{0<X<d\right\}}\right]\right)+\frac{1-S_{X}^{1-\alpha }\left(d\right)}{\alpha -1}$$

$$=-S_{X}^{-\alpha }\left(d\right)E_{f_{X}}\left[\frac{f_{X}^{\alpha -1}\left(x\right)-1}{\alpha -1}I_{\left\{d<X<\infty \right\}}\right]+\frac{1-S_{X}^{1-\alpha }\left(d\right)}{\alpha -1}.$$

We have:

$$E_{f_{X}}\left[\frac{f_{X}^{\alpha -1}\left(x\right)-1}{\alpha -1}I_{\left\{d<X<\infty \right\}}\right]=\frac{1}{\alpha -1}E_{f_{X}}\left[\left({\left(\frac{f_{X}\left(x\right)}{S_{X}\left(x\right)}\right)}^{\alpha -1}\cdot S_{X}^{\alpha -1}\left(x\right)-1\right)I_{\left\{d<X<\infty \right\}}\right]$$

$$=E_{f_{X}}\left[\frac{\lambda^{\alpha -1}\left(x\right)-1}{\alpha -1}\cdot S_{X}^{\alpha -1}\left(x\right)I_{\left\{d<X<\infty \right\}}\right]+E_{f_{X}}\left[\frac{S_{X}^{\alpha -1}\left(x\right)-1}{\alpha -1}I_{\left\{d<X<\infty \right\}}\right]$$

Integrating by parts the second term from the relation above, we obtain:

$$E_{f_{X}}\left[\frac{S_{X}^{\alpha -1}\left(x\right)-1}{\alpha -1}I_{\left\{d<X<\infty \right\}}\right]=\frac{S_{X}^{\alpha }\left(d\right)-S_{X}\left(d\right)}{\alpha -1}-E_{f_{X}}\left[S_{X}^{\alpha -1}\left(x\right)I_{\left\{d<X<\infty \right\}}\right]=$$

$$=\frac{S_{X}^{\alpha }\left(d\right)-S_{X}\left(d\right)}{\alpha -1}-\frac{S_{X}^{\alpha }\left(d\right)}{\alpha }$$

Hence,

$$H_{\alpha }^{T}\left(X_{lt}\left(d\right)\right)=-S_{X}^{-\alpha }\left(d\right)\left[E_{f_{X}}\left[\frac{\lambda^{\alpha -1}\left(x\right)-1}{\alpha -1}\cdot S_{X}^{\alpha -1}\left(x\right)I_{\left\{d<X<\infty \right\}}\right]+\frac{S_{X}^{\alpha }\left(d\right)-S_{X}\left(d\right)}{\alpha -1}-\frac{S_{X}^{\alpha }\left(d\right)}{\alpha }\right]+$$

$$+\frac{1-S_{X}^{1-\alpha }\left(d\right)}{\alpha -1}=-S_{X}^{-\alpha }\left(d\right)E_{f_{X}}\left[\frac{\lambda^{\alpha -1}\left(x\right)-1}{\alpha -1}S_{X}^{\alpha -1}\left(x\right)I_{\left\{d<X<\infty \right\}}\right]+\frac{1}{\alpha }.$$

□

**Theorem 5.**  *Let X be a non-negative random variable which models the loss corresponding to an insurance policy. Let α∈R∖{1}. The Tsallis entropy $H_{\alpha }^{T}\left(X_{lt}\left(d\right)\right)$ of the left-truncated loss random variable corresponding to the per-loss risk model with a deductible d is independent of d if, and only if, the hazard rate function is constant.*

**Proof.**  We assume that the hazard function is constant, therefore λ(x)=k∈R, for any x>0. It results that $f_{X}\left(x\right)=kS_{X}\left(x\right)$, for any x>0 and, using (17), we obtain:

$$H_{\alpha }^{T}\left(X_{lt}\left(d\right)\right)=-S_{X}^{-\alpha }\left(d\right)E_{f_{X}}\left[\frac{\lambda^{\alpha -1}\left(x\right)-1}{\alpha -1}S_{X}^{\alpha -1}\left(x\right)I_{\left\{d<X<\infty \right\}}\right]+\frac{1}{\alpha }$$

$$=\frac{1-k^{\alpha -1}}{\left(\alpha -1\right)S_{X}^{\alpha }\left(d\right)}E_{f_{X}}\left[S_{X}^{\alpha -1}\left(x\right)I_{\left\{d<X<\infty \right\}}\right]+\frac{1}{\alpha }=\frac{1-k^{\alpha -1}}{\alpha (\alpha -1)}+\frac{1}{\alpha }=\frac{\alpha -k^{\alpha -1}}{\alpha (\alpha -1)},$$

which does not depend on *d*.

Conversely, assuming that $H_{\alpha }^{T}\left(X_{lt}\left(d\right)\right)$ does not depend on *d*,

$$\frac{\partial H_{\alpha }^{T}\left(X_{lt}\left(d\right)\right)}{\partial d}=0.$$

Using (17), we obtain

$$-\alpha S_{X}^{\alpha -1}\left(d\right)f\left(d\right)E_{f_{X}}\left[\frac{\lambda^{\alpha -1}\left(x\right)-1}{\alpha -1}S_{X}^{\alpha -1}\left(x\right)I_{\left\{d<X<\infty \right\}}\right]+S_{X}^{-1}\left(d\right)f_{X}\left(d\right)\frac{\lambda^{\alpha -1}\left(d\right)-1}{\alpha -1}=0,$$

i.e.,

$$-S_{X}^{-\alpha }\left(d\right)E_{f_{X}}\left[\frac{\lambda^{\alpha -1}\left(x\right)-1}{\alpha -1}S_{X}^{\alpha -1}\left(x\right)I_{\left\{d<X<\infty \right\}}\right]+\frac{\lambda^{\alpha -1}\left(d\right)-1}{\alpha (\alpha -1)}=0.$$

Using (17) again, the last relation can be expressed as follows:

$$H_{\alpha }^{T}\left(X_{lt}\left(d\right)\right)-\frac{1}{\alpha }+\frac{\lambda^{\alpha -1}\left(d\right)-1}{\alpha (\alpha -1)}=0,$$

which implies

$$\lambda^{\alpha -1}\left(d\right)=\alpha -\alpha \left(\alpha -1\right)H_{\alpha }^{T}\left(X_{lt}\left(d\right)\right)$$

therefore,

$$\lambda \left(d\right)={\left[\alpha -\alpha \left(\alpha -1\right)H_{\alpha }^{T}\left(X_{lt}\left(d\right)\right)\right]}^{\frac{1}{\alpha -1}}.$$

Using again the hypothesis that $H_{\alpha }^{T}\left(X_{lt}\left(d\right)\right)$ does not depend on *d*, it follows that λ does not depend on *d*, therefore λ is constant. □

### 3.2. Loss Models Involving Truncation or Censoring from Above

Right-truncated or censored from below random variables are used in actuarial models with policy limits. In this case, losses are not recorded or reported for or above a specified threshold. We denote by u,u>0 the value of the threshold, referred to as the policy limit or liability limit. According to Kluggman et. al [40], there are two approaches used to express the random variable which models the loss corresponding to the per-payment and per-loss cases, respectively.

In the per-payment case, losses or claims above the value of the liability limit may not be reported to the insurance company, generating truncated from above or right-truncated data.

We denote by $X_{rt}\left(u\right)$ the right-truncated random variable which models the loss corresponding to an insurance policy limit *u* in the per-payment case. It can be expressed as $X_{rt}\left(u\right)=\left[X|X<u\right]$, or equivalently:

(18) $$X_{rt}\left(u\right)=\left\{\begin{array}{c} X,\;if\;\;X<u\\ \mathrm{not}\;\mathrm{defined},\;\;if\;\;X\geq u. \end{array}\right.$$

The relationship between the Tsallis entropy of the random variables *X* and $X_{rt}\left(d\right)$ is established in the following theorem.

**Theorem 6.**  *Let X be a non-negative random variable which models the loss corresponding to an insurance policy. Let α∈R∖{1}. The Tsallis entropy $H_{\alpha }^{T}\left(X_{rt}\left(u\right)\right)$ of the right-truncated loss random variable corresponding to the per-payment risk model with a policy limit u is given by:*

(19) $$H_{\alpha }^{T}\left(X_{rt}\left(u\right)\right)=F_{X}^{-\alpha }\left(u\right)\left(H_{\alpha }^{T}\left(X\right)+E_{f_{X}}\left[\frac{f_{X}{\left(x\right)}^{\alpha -1}-1}{\alpha -1}I_{\left\{u<X<\infty \right\}}\right]\right)+\frac{1-F_{X}^{1-\alpha }\left(u\right)}{\alpha -1}.$$

**Proof.**  The probability density function of the random variable $X_{rt}\left(u\right)$ is given by $f_{X_{rt}\left(u\right)}\left(x\right)=\frac{f_{X}\left(x\right)}{F_{X}\left(u\right)}$, 0<x<u. Therefore, the Tsallis entropy of the random variable $X_{rt}\left(u\right)$ can be expressed as follows:

$$H_{\alpha }^{T}\left(X_{rt}\left(u\right)\right)=-\frac{1}{\left(\alpha -1\right)F_{X}\left(u\right)}E_{f_{X}}\left[\left({\left(\frac{f_{X}\left(x\right)}{F_{X}\left(u\right)}\right)}^{\alpha -1}-1\right)I_{\left\{0<X<u\right\}}\right]=$$

$$=-F_{X}^{-\alpha }\left(u\right)E_{f_{X}}\left[\frac{f_{X}^{\alpha -1}\left(x\right)-1}{\alpha -1}I_{\left\{0<X<u\right\}}\right]+\frac{1-F_{X}^{1-\alpha }\left(u\right)}{\left(\alpha -1\right)F_{X}\left(u\right)}E_{f_{X}}\left[I_{\left\{0<X<u\right\}}\right]=$$

$$=-F_{X}^{-\alpha }\left(u\right)E_{f_{X}}\left[\frac{f_{X}^{\alpha -1}\left(x\right)-1}{\alpha -1}I_{\left\{0<X<u\right\}}\right]+\frac{1-F_{X}^{1-\alpha }\left(u\right)}{\alpha -1}$$

$$=F_{X}^{-\alpha }\left(u\right)\left(-E_{f_{X}}\left[\frac{f_{X}^{\alpha -1}\left(x\right)-1}{\alpha -1}\right]+E_{f_{X}}\left[\frac{f_{X}^{\alpha -1}\left(x\right)-1}{\alpha -1}I_{\left\{u<X<\infty \right\}}\right]\right)+\frac{1-F_{X}^{1-\alpha }\left(u\right)}{\alpha -1}=$$

$$=F_{X}^{-\alpha }\left(u\right)\left(H_{\alpha }^{T}\left(X\right)+E_{f_{X}}\left[\frac{f_{X}^{\alpha -1}\left(x\right)-1}{\alpha -1}I_{\left\{u<X<\infty \right\}}\right]\right)+\frac{1-F_{X}^{1-\alpha }\left(u\right)}{\alpha -1}.$$

□

In the following theorem, the Tsallis entropy of the right-truncated loss random variable corresponding to the per-payment risk model with a policy limit is derived.

**Theorem 7.**  *Let X be a non-negative random variable which models the loss corresponding to an insurance policy. Let α∈R∖{1} and u>0. The Tsallis entropy $H_{\alpha }^{T}\left(X_{rt}\left(u\right)\right)$ of the right-truncated loss random variable corresponding to the per-payment risk model with a policy limit u can be expressed in terms of the reversed hazard function as follows:*

(20) $$H_{\alpha }^{T}\left(X_{rt}\left(u\right)\right)=-F_{X}^{-\alpha }\left(u\right)E_{f_{X}}\left[\frac{\tau^{\alpha -1}\left(x\right)-1}{\alpha -1}F_{X}^{\alpha -1}\left(x\right)I_{\left\{0<X<u\right\}}\right]-$$

(21) $$-F_{X}^{-1}\left(u\right)E_{f_{X}}\left[\frac{F_{X}^{\alpha -1}\left(x\right)-1}{\alpha -1}I_{\left\{0<X<u\right\}}\right]+\frac{1-F_{X}^{1-\alpha }\left(u\right)}{\alpha -1}.$$

**Proof.**  The probability density function of the random variable $X_{rt}\left(u\right)$ is given by $f_{X_{rt}\left(u\right)}\left(x\right)=\frac{f_{X}\left(x\right)}{F_{X}\left(u\right)}$, 0<x<u. Therefore, the Tsallis entropy of the random variable $X_{rt}\left(u\right)$ can be expressed as follows:

$$H_{\alpha }^{T}\left(X_{rt}\left(u\right)\right)=\frac{1}{\left(\alpha -1\right)F_{X}\left(u\right)}E_{f_{X}}\left[\left({\left(\frac{f_{X}\left(x\right)}{F_{X}\left(u\right)}\right)}^{\alpha -1}-1\right)I_{\left\{0<X<u\right\}}\right]=$$

$$=-\frac{1}{\left(\alpha -1\right)F_{X}\left(u\right)}\cdot$$

$$E_{f_{X}}\left[\left({\left(\frac{f_{X}\left(x\right)}{F_{X}\left(x\right)}\right)}^{\alpha -1}{\left(\frac{F_{X}\left(x\right)}{F_{X}\left(u\right)}\right)}^{\alpha -1}-{\left(\frac{F_{X}\left(x\right)}{F_{X}\left(u\right)}\right)}^{\alpha -1}+{\left(\frac{F_{X}\left(x\right)}{F_{X}\left(u\right)}\right)}^{\alpha -1}-1\right)I_{\left\{0<X<u\right\}}\right]=$$

$$=-\frac{1}{\left(\alpha -1\right)F_{X}\left(u\right)}E_{f_{X}}\left[{\left(\frac{F_{X}\left(x\right)}{F_{X}\left(u\right)}\right)}^{\alpha -1}\left({\left(\frac{f_{X}\left(x\right)}{F_{X}\left(x\right)}\right)}^{\alpha -1}-1\right)I_{\left\{0<X<u\right\}}\right]-$$

$$-\frac{1}{\left(\alpha -1\right)F_{X}\left(u\right)}E_{f_{X}}\left[\left({\left(\frac{F_{X}\left(X\right)}{F_{X}\left(u\right)}\right)}^{\alpha -1}-1\right)I_{\left\{0<X<u\right\}}\right]$$

$$=-\frac{F_{X}^{-\alpha }\left(u\right)}{\alpha -1}E_{f_{X}}\left[F_{X}^{\alpha -1}\left(x\right)\left({\left(\frac{f_{X}\left(x\right)}{F_{X}\left(x\right)}\right)}^{\alpha -1}-1\right)I_{\left\{0<X<u\right\}}\right]$$

$$-\frac{1}{\left(\alpha -1\right)F_{X}\left(u\right)}E_{f_{X}}\left[\left({\left(\frac{F_{X}\left(x\right)}{F_{X}\left(u\right)}\right)}^{\alpha -1}-F_{X}^{1-\alpha }\left(u\right)+F_{X}^{1-\alpha }\left(u\right)-1\right)I_{\left\{0<X<u\right\}}\right]$$

$$=-\frac{F_{X}^{-\alpha }\left(u\right)}{\alpha -1}E_{f_{X}}\left[F_{X}^{\alpha -1}\left(x\right)\left({\left(\frac{f_{X}\left(x\right)}{F_{X}\left(x\right)}\right)}^{\alpha -1}-1\right)I_{\left\{0<X<u\right\}}\right]-$$

$$-F_{X}^{-\alpha }\left(u\right)E_{f_{X}}\left[\frac{F_{X}^{\alpha -1}\left(x\right)-1}{\alpha -1}I_{\left\{0<X<u\right\}}\right]+\frac{1-F_{X}^{1-\alpha }\left(u\right)}{\left(\alpha -1\right)F_{X}\left(u\right)}E_{f_{X}}\left[I_{\left\{0<X<u\right\}}\right]=$$

$$=-F_{X}^{-\alpha }\left(u\right)E_{f_{X}}\left[\frac{\tau^{\alpha -1}\left(x\right)-1}{\alpha -1}F_{X}^{\alpha -1}\left(x\right)I_{\left\{0<X<u\right\}}\right]-$$

$$-F_{X}^{-\alpha }\left(u\right)E_{f_{X}}\left[\frac{F_{X}^{\alpha -1}\left(x\right)-1}{\alpha -1}I_{\left\{0<X<u\right\}}\right]+\frac{1-F_{X}^{1-\alpha }\left(u\right)}{\alpha -1}.$$

□

Now, we consider the case of the per-loss right censoring. In this case, if the loss exceeds the value of the policy limit, the insurance company pays an amount *u*.

For example, a car insurance policy covers losses up to a limit *u*, while major losses are covered by the car owner. If the loss is modeled by the random variable *X*, then the loss corresponding to the insurance company is represented by $\left[X|X<u\right]$. We note that the loss model with truncation from above is different from the loss model with censoring from above, which is defined by the random variable $X_{rc}\left(u\right)=\min\left\{X,\;u\right\}$. In this case, if the loss is X≥u, the insurance company pays an amount *u*.

The loss model with censoring from above is modeled using the random variable $X_{rc}\left(u\right)=\min\left\{X,\;u\right\}$. Moreover, it can be represented as

(22) $$X_{rc}\left(u\right)=\left\{\begin{array}{c} X,\;if\;\;X<u\\ u,\;\;if\;\;X\geq u. \end{array}\right.$$

This model, corresponding to the per-loss case, assumes that in the case where the loss is X≥u, the insurance company pays an amount *u*. Therefore, the insurer pays a maximum amount of *u* on a claim. We note that the random variable $X_{rc}\left(u\right)$ is not absolutely continuous.

In the following theorem, an analytical formula for the entropy corresponding to the random variable $X_{rc}\left(u\right)$ is obtained.

**Theorem 8.**  *Let X be a non-negative random variable which models the loss corresponding to an insurance policy. Let α∈R∖{1} and u>0. The Tsallis entropy of losses for the right-censored loss random variable corresponding to the per-payment risk model with a policy limit u can be expressed as follows:*

(23) $$H_{\alpha }^{T}\left(X_{rc}\left(u\right)\right)=H_{\alpha }^{T}\left(X\right)+E_{f_{X}}\left[\frac{f_{X}{\left(x\right)}^{\alpha -1}-1}{\alpha -1}I_{\left\{u<X<\infty \right\}}\right]-S_{X}\left(u\right)\frac{S_{X}{\left(u\right)}^{\alpha -1}-1}{\alpha -1}.$$

**Proof.**  We have:

$$H_{\alpha }^{T}\left(X_{rc}\left(u\right)\right)=-E_{f_{X}}\left[\frac{f_{X}^{\alpha -1}\left(x\right)-1}{\alpha -1}I_{\left\{0<X<u\right\}}\right]-S_{X}\left(u\right)\frac{S_{X}^{\alpha -1}\left(u\right)-1}{\alpha -1}$$

$$=H_{\alpha }^{T}\left(X\right)+E_{f_{X}}\left[\frac{f_{X}^{\alpha -1}\left(x\right)-1}{\alpha -1}I_{\left\{u<X<\infty \right\}}\right]-S_{X}\left(u\right)\frac{S_{X}^{\alpha -1}\left(u\right)-1}{\alpha -1}.$$

□

### 3.3. Loss Models Involving Truncation from Above and from Below

We denote by *d* the deductible and by *u* the retention limit, with d<u. The deductible is applied after the implementation of the retention limit *u*. Therefore, if the value of the loss is grater than *u*, then the value of the maximum payment is u−d. We denote by $X_{lr}\left(d,\;u\right)$ the loss random variable which models the payments to the policy holder under a combination of deductible and retention limit policies. $X_{lr}\left(d,\;u\right)$ is a mixed random variable, with an absolutely continuous part over the interval (0,u−d) and two discrete parts at 0, with probability mass $F_{X}\left(d\right)$ and at u−d and with probability mass $S_{X}\left(u\right)$. Following [40], the loss random variable $X_{lr}\left(d,\;u\right)$ can be expressed by:

(24) $$X_{lr}\left(d,\;u\right)=\left\{\begin{array}{c} 0,\;if\;\;X\leq d\\ X-d,\;if\;\;d<X\leq u\\ u-d,\;\;if\;\;X>u, \end{array}\right.$$

The deductible *d* is applied after the implementation of the retention limit *u*, which means that if the loss is greater than *u*, then the maximum payment is u−d. The random variable $X_{lr}\left(d,u\right)$ is a mixed variable with an absolutely continuous part over the interval (0,u−d) and two discrete parts at 0, with probability mass $F_{X}\left(d\right)$ and at u−d and with probability mass $S_{X}\left(u\right)$.

In the next theorem, the Tsallis entropy of losses for the right-truncated loss random variable corresponding to the per-loss risk model with a deductible *d* and a policy limit *u* is derived.

**Theorem 9.**  *Let X be a non-negative random variable which models the loss corresponding to an insurance policy. Let α∈R∖{1}, d>0 and u>d. The Tsallis entropy of losses of the right-truncated loss random variable corresponding to the per-loss risk model with a deductible d and a policy limit u is given by:*

$$H_{\alpha }^{T}\left(X_{lr}\left(d,\;u\right)\right)=H_{\alpha }^{T}\left(X\right)+E_{f_{X}}\left[\frac{f_{X}{\left(x\right)}^{\alpha -1}-1}{\alpha -1}I_{\left\{0<X<d\right\}}\right]+E_{f_{X}}\left[\frac{f_{X}{\left(x\right)}^{\alpha -1}-1}{\alpha -1}I_{\left\{u<X<\infty \right\}}\right]-$$

(25) $$-F_{X}\left(d\right)\frac{F_{X}{\left(d\right)}^{\alpha -1}-1}{\alpha -1}-S_{X}\left(u\right)\frac{S_{X}{\left(u\right)}^{\alpha -1}-1}{\alpha -1}$$

**Proof.**  The probability density function of the random variable $X_{lr}\left(d,u\right)$ is given by

(26) $$f_{X_{lr}\left(d,u\right)}\left(x\right)=F_{X}\left(d\right)\delta_{\left\{x=0\right\}}+f_{X}\left(x+d\right)\delta_{\left\{u<X<u-d\right\}}+S_{X}\left(u\right)\delta_{\left\{x=u-d\right\}}$$

where δ denotes the Dirac delta function.

It results:

$$H_{\alpha }^{T}\left(X_{lr}\left(d,u\right)\right)=-F_{X}\left(d\right)\frac{F_{X}^{\alpha -1}\left(d\right)-1}{\alpha -1}-E_{f_{X}}\left[\frac{f_{X}^{\alpha -1}\left(x\right)-1}{\alpha -1}I_{\left\{d<X<u\right\}}\right]-S_{X}\left(u\right)\frac{S_{X}^{\alpha -1}\left(u\right)-1}{\alpha -1}$$

$$=H_{\alpha }^{T}\left(X\right)+E_{f_{X}}\left[\frac{f_{X}^{\alpha -1}\left(x\right)-1}{\alpha -1}I_{\left\{0<X<d\right\}}\right]+E_{f_{X}}\left[\frac{f_{X}^{\alpha -1}\left(x\right)-1}{\alpha -1}I_{\left\{u<X<\infty \right\}}\right]-$$

$$-F_{X}\left(d\right)\frac{F_{X}^{\alpha -1}\left(d\right)-1}{\alpha -1}-S_{X}\left(u\right)\frac{S_{X}^{\alpha -1}\left(u\right)-1}{\alpha -1}.$$

□

The following theorem establishes the relationship between $H_{\alpha }^{T}\left(X_{lr}\left(d,u\right)\right)$, the entropy under censoring from above $H_{\alpha }^{T}\left(X_{rc}\left(u\right)\right)$ and the entropy under censoring from below $H_{\alpha }^{T}\left(X_{lc}\left(d\right)\right)$.

**Theorem 10.**  *Let X be a non-negative random variable which models the loss corresponding to an insurance policy. Let α∈R∖{1}. For any d>0 and u>d, the Tsallis entropy $H_{\alpha }^{T}\left(X_{lr}\left(d,u\right)\right)$ is related to the entropies $H_{\alpha }^{T}\left(X_{rc}\left(u\right)\right)$ and $H_{\alpha }^{T}\left(X_{lc}\left(d\right)\right)$ through the following relationship:*

$$\begin{array}{ccc} H_{sl}\left(f_{X},d,u\right) & = & -F_{X}\left(d\right)\frac{F_{X}^{\alpha -1}\left(d\right)-1}{\alpha -1}+E_{f_{X}}\left[\frac{f_{X}^{\alpha -1}\left(x\right)-1}{\alpha -1}I_{\left\{0<X<d\right\}}\right]-S_{X}\left(u\right)\frac{S_{X}^{\alpha -1}\left(u\right)-1}{\alpha -1}\\ & & +F_{X}^{\alpha }\left(u\right)H_{\alpha }^{T}\left(X_{rc}\left(u\right)\right)-F_{X}\left(u\right)\frac{F_{X}^{\alpha -1}\left(u\right)-1}{\alpha -1}. \end{array}$$

**Proof.**  We have:

$$H_{\alpha }^{T}\left(X_{lr}\left(d,u\right)\right)=H_{\alpha }^{T}\left(X\right)+E_{f_{X}}\left[\frac{f_{X}^{\alpha -1}\left(x\right)-1}{\alpha -1}I_{\left\{0<X<d\right\}}\right]+E_{f_{X}}\left[\frac{f_{X}^{\alpha -1}\left(x\right)-1}{\alpha -1}I_{\left\{u<X<\infty \right\}}\right]-$$

$$-F_{X}\left(d\right)\frac{F_{X}^{\alpha -1}\left(d\right)-1}{\alpha -1}-S_{X}\left(u\right)\frac{S_{X}^{\alpha -1}\left(u\right)-1}{\alpha -1}.$$

Moreover,

$$H_{\alpha }^{T}\left(X_{rc}\left(u\right)\right)=H_{\alpha }^{T}\left(X\right)+E_{f_{X}}\left[\frac{f_{X}^{\alpha -1}\left(x\right)-1}{\alpha -1}I_{\left\{u<X<\infty \right\}}\right]-S_{X}\left(u\right)\frac{S_{X}^{\alpha -1}\left(u\right)-1}{\alpha -1}$$

$$H_{\alpha }^{T}\left(X_{lc}\left(d\right)\right)=H_{\alpha }^{T}\left(X\right)+E_{f_{X}}\left[\frac{f_{X}^{\alpha -1}\left(x\right)-1}{\alpha -1}I_{\left\{0<X<d\right\}}\right]-F_{X}\left(d\right)\frac{F_{X}^{\alpha -1}\left(d\right)-1}{\alpha -1}.$$

It results that:

$$H_{sl}\left(f,d,u\right)=H_{\alpha }^{T}\left(X_{rc}\left(u\right)\right)-F_{X}\left(d\right)\frac{F_{x}^{\alpha -1}\left(d\right)-1}{\alpha -1}+E_{f_{X}}\left[\frac{f_{X}^{\alpha -1}\left(x\right)-1}{\alpha -1}I_{\left\{0<X<d\right\}}\right]=$$

$$=-F_{X}\left(d\right)\frac{F_{X}^{\alpha -1}\left(d\right)-1}{\alpha -1}+E_{f_{X}}\left[\frac{f_{x}^{\alpha -1}\left(x\right)-1}{\alpha -1}I_{\left\{0<X<d\right\}}\right]-$$

$$-S_{X}\left(u\right)\frac{S_{X}^{\alpha -1}\left(u\right)-1}{\alpha -1}+F_{X}^{\alpha }\left(u\right)H_{\alpha }^{T}\left(X_{rc}\left(u\right)\right)-F_{X}\left(u\right)\frac{F_{X}^{\alpha -1}\left(u\right)-1}{\alpha -1}.$$

□

**Figure 2:**
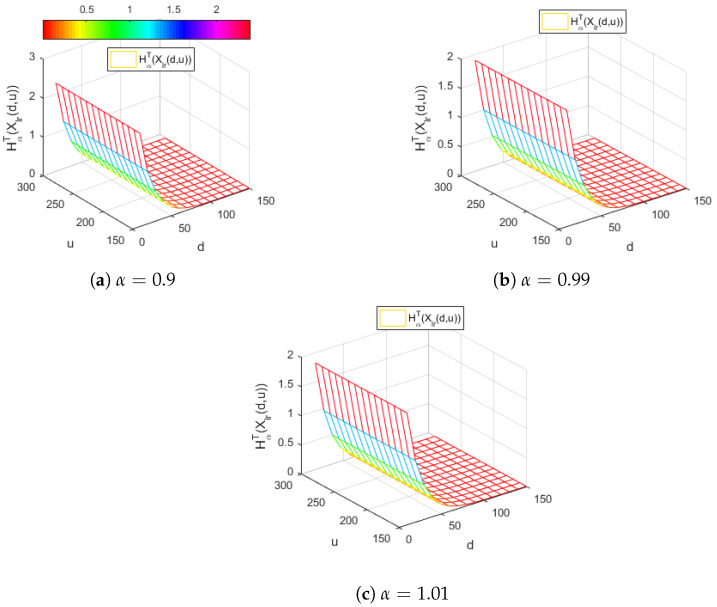
The Tsallis entropy $H_{\alpha }^{T}\left(X_{lr}\left(d,\;u\right)\right)$ of losses for the right-truncated loss random variable corresponding to the per-loss risk model with a deductible *d* and a policy limit *u* for the exponential distribution, with λ=0.1 and different values of the Tsallis parameter α.

Figure 2 illustrates the Tsallis entropy of the right-truncated loss random variable $X_{lr}\left(d,\;u\right)$, corresponding to the per-loss risk model with a deductible *d* and a policy limit *u* for the exponential distribution with λ=0.1.

Figure 2 displays a similar behavior of the Tsallis entropy $H_{\alpha }^{T}\left(X_{lr}\left(d,\;u\right)\right)$ for all the considered values around 1 of the α parameter. Thus, we remark that, for all values of α, the Tsallis entropy $H_{\alpha }^{T}\left(X_{lr}\left(d,\;u\right)\right)$ is decreasing with respect to the deductible *d* and it does not depend on the policy limit *u*.

**Figure 3:**
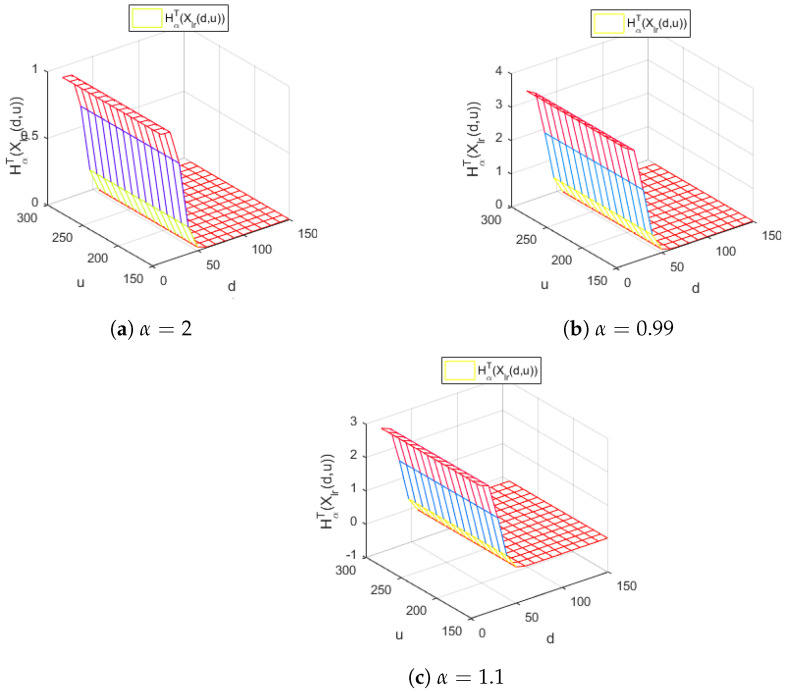
The Tsallis entropy of $H_{\alpha }^{T}\left(X_{lr}\left(d,\;u\right)\right)$ losses of the right-truncated loss random variable corresponding to the per-loss risk model with a deductible *d* and a policy limit *u* for the $\chi^{2}$ distribution, with γ=30 and different values of the Tsallis parameter α.

Figure 3 represents the Tsallis entropy of losses for the right-truncated loss random variable $X_{lr}\left(d,\;u\right)$ corresponding to the per-loss risk model with a deductible *d* and a policy limit *u* for the $\chi^{2}$ distribution, with γ=30 and for different values of the Tsallis parameter α, in the case d<u.

Figure 3 reveals, for all the values of the parameter α considered, a similar decreasing behavior with respect to the deductible *d* of the Tsallis entropy $H_{\alpha }^{T}\left(X_{lr}\left(d,\;u\right)\right)$. Moreover, it indicates that the Tsallis entropy $H_{\alpha }^{T}\left(X_{lr}\left(d,\;u\right)\right)$ does not depend on the values of the policy limit *u*.

**Figure 4:**
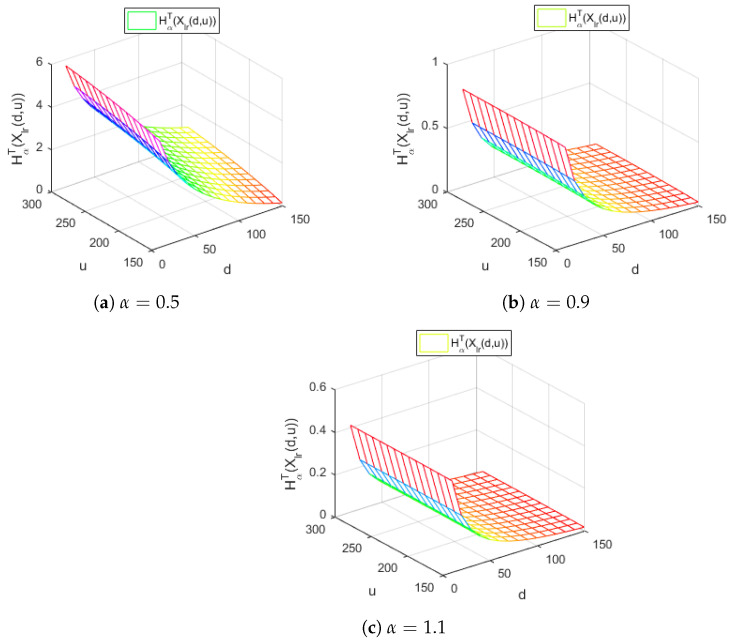
The Tsallis entropy of losses $H_{\alpha }^{T}\left(X_{lr}\left(d,\;u\right)\right)$ of the right-truncated loss random variable corresponding to the per-loss risk model with a deductible *d* and a policy limit *u* for the Weibull distribution, with γ=0.3,λ=1.3,a=0 and different values of the Tsallis parameter α.

Figure 4 depicts the Tsallis entropy of losses of the right-truncated loss random variable corresponding to the per-loss risk model with a deductible *d* and a policy limit *u* for the Weibull distribution, with γ=0.3,λ=1.3 and a=0 for different values of the Tsallis parameter α, in the case d<u.

Figure 4 highlights that the Tsallis entropy of losses $H_{\alpha }^{T}\left(X_{lr}\left(d,\;u\right)\right)$ is decreasing with respect to *d* for all the values of the parameter α considered. Moreover, the Tsallis entropy $H_{\alpha }^{T}\left(X_{lr}\left(d,\;u\right)\right)$ does not depend on the policy limit *u* for the values of the α parameter around 1, respectively, for α=0.9 and α=1.1. A different behavior is detected for α=0.5. In this case, we remark that the Tsallis entropy is increasing with respect to the policy limit *u*, which is realistic from the actuarial point of view. Indeed, increasing the policy limit results in a higher risk for the insurance company.

The conclusions obtained indicate that Tsallis entropy measures with parameter values significantly different from 1 can provide a better loss model involving truncation from above and from below.

**Figure 5:**
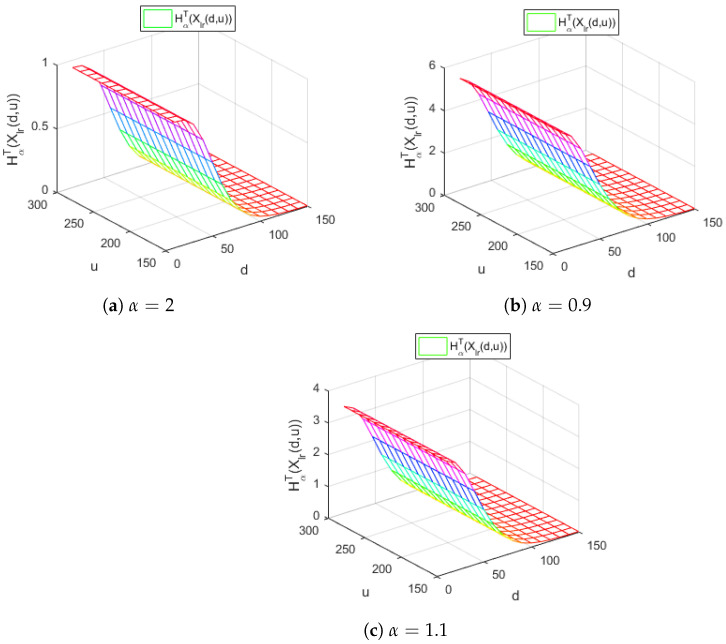
The Tsallis entropy $H_{\alpha }^{T}\left(X_{lr}\left(d,\;u\right)\right)$ of losses of the right-truncated loss random variable corresponding to the per-loss risk model with a deductible *d* and a policy limit *u* for the Gamma distribution, with γ=4.5,λ=0.1,a=0.01 and different values of the Tsallis parameter α.

Figure 5 displays the Tsallis entropy of losses for the right-truncated loss random variable corresponding to the per-loss risk model with a deductible *d* and a policy limit *u* for the Gamma distribution, with γ=4.5,λ=0.1 and a=0.01 for different values of the Tsallis parameter α, in the case d<u.

Figure 5 reveals the decreasing behavior of the Tsallis entropy $H_{\alpha }^{T}\left(X_{lr}\left(d,\;u\right)\right)$ of losses for all the values of the Tsallis parameter α considered. Moreover, for all the values of α, the Tsallis entropy $H_{\alpha }^{T}\left(X_{lr}\left(d,\;u\right)\right)$ does not depend on the policy limit *u*.

The following tables present the Tsallis entropy values for the Weibull distribution, corresponding to the analyzed models.

Table 1 illustrates the Tsallis entropy values in case of the Weibull distribution with λ=0.9585,γ=0.3192 and d=1.1 for different values of the Tsallis parameter α and several values of the policy limit *u*.

The analysis of the results presented in Table 1 reveals that for parameter values α≠1 the Tsallis entropy corresponding to the $X_{rt}\left(u\right)$ random variable is increasing with respect to the value of the policy limit *u*. On the other side, for α=1, the Tsallis entropy, which reduces to the Shannon entropy measure, is decreasing with respect to *u*. From an actuarial perspective, when the policy limit increases, the risk of the insurance company also increases, therefore the entropy of losses increases, too. The detected behavior of the Tsallis entropy measure is reasonable in this case, and it means that the Tsallis entropy approach for evaluating the risk corresponding to the $X_{rt}\left(u\right)$ random variable is more realistic.

Table 2 displays the values of the Tsallis entropy measures in case of the Weibull distribution with λ=0.9585,γ=0.3192 and d=1.1 for different values of the Tsallis parameter α and several values of the policy limit *u*.

Analyzing the results presented in Table 2, we remark that for parameter values α≠1 the Tsallis entropy corresponding to the $X_{rt}\left(u\right)$ random variable is increasing with respect to the value of the policy limit *u*. On the other side, for α=1, the Tsallis entropy, which reduces to the Shannon entropy measure, is decreasing with respect to *u*. From an actuarial perspective, when the policy limit increases, the risk of the insurance company also increases, therefore the entropy of losses increases, too. The detected behavior of the Tsallis entropy measure is reasonable in this case, and it means that the Tsallis entropy approach for evaluating the risk corresponding to the $X_{rt}\left(u\right)$ random variable is more realistic.

Table 3 illustrates the Tsallis entropy values in the case of the Weibull distribution with λ=0.9585,γ=0.3192 and deductible d=1.2 for various values of the Tsallis parameter α and several values of the policy limit *u*.

The study of the results presented in Table 3 reveals that for parameter values α≠1 the Tsallis entropy corresponding to the $X_{rt}\left(u\right)$ random variable is increasing with respect to the value of the policy limit *u*. On the other side, for α=1, the Tsallis entropy, which reduces to the Shannon entropy measure, is decreasing with respect to *u*. From an actuarial perspective, when the policy limit increases, the risk of the insurance company also increases, therefore the entropy of losses increases, too. The detected behavior of the Tsallis entropy measure is reasonable in this case, and it means that the Tsallis entropy approach for evaluating the risk corresponding to the $X_{rt}\left(u\right)$ random variable is more realistic.

Table 4 reveals the values of all the Tsallis entropy measures analyzed in the case of the Weibull distribution with λ=0.9585,γ=0.3192 and d=1.3 for several values of the Tsallis parameter α and different values of the policy limit *u*.

The results displayed in Table 4 show that for α≠1 the Tsallis entropy of the $X_{rt}\left(u\right)$ random variable increases with respect to the value of the policy limit *u*, whereas for α=1, the entropy decreases with respect to *u*. It indicates that, when the policy limit increases, the risk of the insurance company increases, too. Thus, the entropy of losses is increasing. We can also conclude that in this case the right-truncated loss random variable $X_{rt}\left(u\right)$ is better modeled using Tsallis entropy measure.

Table 5 displays the Tsallis entropy values in case of the Weibull distribution with λ=0.9585,γ=0.3192 and deductible d=1.4 for different values of the Tsallis parameter α and several values of the policy limit *u*.

Analyzing the results provided in Table 5, we remark that for the parameter α≠1 the Tsallis entropy corresponding to the right-truncated random variable is increasing with respect to the value of the policy limit *u*. For α=1, the Shannon entropy measure decreases with respect to *u*. From an actuarial perspective, when the policy limit increases, the risk of the insurance company also increases, therefore the entropy of losses increases, too. The detected behavior of the Tsallis entropy measure is reasonable in this case, and it means that the Tsallis entropy approach for evaluating the risk corresponding to the $X_{rt}\left(u\right)$ random variable is more realistic.

From Table 1, Table 2, Table 3, Table 4 and Table 5, we draw the following conclusions. Using the Tsallis entropy measure approach, in the case when the deductible value *d* increases, the uncertainty of losses for the insurance company will decrease, therefore the company has to pay smaller amounts. In the case when the policy limit value *u* increases, the uncertainty of losses for the insurance company will increase, as the company has to pay greater amounts. Therefore, the Tsallis entropy approach is more realistic and flexible, providing a relevant perspective and a useful instrument for loss models.

### 3.4. Loss Models under Inflation

Financial and actuarial models are estimated using observations made in the past years. As inflation implies an increase in losses, the models must be adjusted corresponding to the current level of loss experience. Moreover, a projection of the anticipated losses in the future needs to be performed.

Now, we study the effect of inflation on entropy. Let *X* be the random variable that models the loss corresponding to a certain year. We denote by *F* the cumulative distribution function of *X* and by *f* the probability density function of *X*. The random variable that models the loss after one year and under the inflation effect is X(r)=(1+r)X, where r,r>0, represents the annual inflation rate. We denote by $F_{X(r)}$ the cumulative distribution function of X(r) and by $f_{X(r)}$ the probability density function of the random variable X(r).

The probability density function corresponding to the random variable X(r) is given by:

$$f_{X(r)}\left(z\right)=\frac{1}{1+r}f_{X}\left(\frac{z}{1+r}\right),\;z\in R.$$

The following theorem derives the relationship between the Tsallis entropies of the random variables *X* and X(r)=(1+r)X.

**Theorem 11.**  *Let X be a non-negative random variable which models the loss corresponding to an insurance policy. Let α∈R∖{1}. The Tsallis entropy of the random variable X(r), which models the loss after one year under inflation rate r, r>0, is given by*

(27) $$H_{\alpha }^{T}\left(X(r)\right)={\left(1+r\right)}^{1-\alpha }H_{\alpha }^{T}\left(X\right)-\frac{{\left(1+r\right)}^{1-\alpha }-1}{\alpha -1}.$$

**Proof.**  Using the definition of the Tsallis entropy, we have:

$$H_{\alpha }^{T}\left(X(r)\right)=-E_{f_{X}}\left[\frac{f_{X(r)}^{\alpha -1}\left(x\right)-1}{\alpha -1}\right].$$

Using the change in variable given by $u=\frac{z}{1+r}$, it follows

$$H_{\alpha }^{T}\left(X(r)\right)={\left(1+r\right)}^{1-\alpha }E_{f_{X}}\left[\frac{f_{X}^{\alpha -1}\left(x\right)-1}{\alpha -1}\right]-\frac{{\left(1+r\right)}^{1-\alpha }-1}{\alpha -1}=$$

$$={\left(1+r\right)}^{1-\alpha }H_{\alpha }^{T}\left(X\right)-\frac{{\left(1+r\right)}^{1-\alpha }-1}{\alpha -1}$$

□

**Theorem 12.**  *Let X be a non-negative random variable which models the loss corresponding to an insurance policy. Let α∈R∖{1}. For r>0, the Tsallis entropy of the random variable X(r), which models the loss after one year under inflation rate r,r>0, is always larger than that of X and is an increasing function of r.*

**Proof.**  Let r>0. We denote by

$$\psi \left(r\right)=H_{\alpha }^{T}\left(X(r)\right)-H_{\alpha }^{T}\left(X\right)$$

$$={\left(1+r\right)}^{1-\alpha }H_{\alpha }^{T}\left(X\right)-H_{\alpha }^{T}\left(X\right)-\frac{{\left(1+r\right)}^{1-\alpha }-1}{\alpha -1}=\left[{\left(1+r\right)}^{1-\alpha }-1\right]\left(H_{\alpha }^{T}\left(X\right)-\frac{1}{\alpha -1}\right)$$

We have:

$$\frac{d}{dr}\psi \left(r\right)={\left(1+r\right)}^{2-\alpha }\left[\left(\alpha -1\right)H_{\alpha }^{T}\left(X\right)-1\right]$$

$$={\left(1+r\right)}^{2-\alpha }E_{f_{X}}\left[f_{X}^{\alpha -1}\left(x\right)\right]>0,$$

so that $H_{\alpha }^{T}\left(X(r)\right)$ is an increasing function of *r*.

Therefore, it follows that

$$H_{\alpha }^{T}\left(X(r)\right)>H_{\alpha }^{T}\left(X\right).$$

□

The results obtained show that inflation increases the entropy, which means that the uncertainty degree of losses increases compared with the case without inflation. Moreover, the uncertainty of losses increases with respect to the inflation rate.

## 4. Tsallis Entropy Approach for Survival Models

In this section, we derive residual and past entropy expressions for some survival models, including the proportional hazard and the proportional reversed hazard models. Relevant results in this field have been obtained by Sachlas and Papaioannou [18], Gupta and Gupta [19], Di Crescenzo [41] and Sankaran and Gleeja [42].

Let *X* and *Y* be random variables with cumulative distribution functions *F* and *G*, probability density functions *f* and *g* and survival functions $\bar{F}$ and $\bar{G}$, respectively. We denote by $\lambda_{X}$ and $\lambda_{Y}$ the hazard rate functions of the random variables *X* and *Y*, respectively.

### 4.1. The Proportional Hazard Rate Model

**Definition 3.**  *The random variables X and Y satisfy the proportional hazard rate model if there exists θ>0 such that (see Cox* [43]*).*

(28) $$S_{Y}\left(x\right)=S_{X}^{\theta }\left(x\right)\;for\;every\;x>0.$$

We note that the random variables *X* and *Y* satisfy the proportional hazard rate model if the hazard rate function of *Y* is proportional to the hazard rate function of *X*, i.e., $\lambda_{{}_{Y}}\left(x\right)=\theta \lambda_{{}_{X}}\left(x\right)$ for every x>0; see Cox [43].

In the next theorem, the Tsallis entropy of the left-truncated random variable $Y_{lt}\left(d\right)$ under the proportional hazard rate model is derived.

**Theorem 13.**  *Let X and Y be non-negative random variables. Let α∈R∖{1} and d>0. Under the proportional hazard rate model given in (28), the Tsallis entropy of the left-truncated random variable $Y_{lt}\left(d\right)$ corresponding to the per-payment risk model with a deductible d can be expressed as follows:*

(29) $$H_{\alpha }^{T}\left(Y_{lt}\left(d\right)\right)=-\frac{\theta }{\left(\alpha -1\right)S_{X}^{\theta }\left(d\right)}E_{f_{X}}\left[S_{X}^{\theta -1}\left(x\right)\left({\left(\frac{\theta S_{X}^{\theta -1}\left(x\right)f_{X}\left(x\right)}{S_{X}^{\theta }\left(d\right)}\right)}^{\alpha -1}-1\right)I_{\left\{d<X<\infty \right\}}\right].$$

**Proof.**  From (28), we obtain $f_{Y}\left(x\right)=\theta S_{X}^{\theta -1}\left(x\right)f_{X}\left(x\right)$. It results:

$$H_{\alpha }^{T}\left(Y_{lt}\left(d\right)\right)=-\frac{1}{\left(\alpha -1\right)S_{Y}\left(d\right)}E_{f_{Y}}\left[\left({\left(\frac{f_{Y}\left(x\right)}{S_{Y}\left(d\right)}\right)}^{\alpha -1}-1\right)I_{\left\{d<X<\infty \right\}}\right]$$

$$=-\frac{\theta }{\left(\alpha -1\right)S_{X}^{\theta }\left(d\right)}E_{f_{X}}\left[S_{X}^{\theta -1}\left(x\right)\left({\left(\frac{\theta S_{X}^{\theta -1}\left(x\right)f_{X}\left(x\right)}{S_{X}^{\theta }\left(d\right)}\right)}^{\alpha -1}-1\right)I_{\left\{d<X<\infty \right\}}\right].$$

□

### 4.2. The Proportional Reversed Hazard Rate Model

**Definition 4.**  *The random variables X and Y satisfy the proportional reversed hazard rate model* [43] *if there exists θ>0 such that*

(30) $$F_{Y}\left(x\right)=F_{X}^{\theta }\left(x\right)\;for\;every\;x>0.$$

In the next theorem, the Tsallis entropy of the right-truncated random variable $Y_{rt}\left(u\right)$ under the proportional reversed hazard rate model is derived.

**Theorem 14.**  *Let X and Y be non-negative random variables. Let α∈R∖{1} and u>0. Under the proportional reversed hazard rate model given in (30), the Tsallis entropy of the right-truncated random variable $Y_{rt}\left(u\right)$ corresponding to the per-payment risk model with a policy limit u can be expressed as follows:*

(31) $$H_{\alpha }^{T}\left(Y_{rt}\left(d\right)\right)=-\frac{\theta }{\left(\alpha -1\right)F_{X}^{\theta }\left(u\right)}E_{f_{X}}\left[F_{X}^{\theta -1}\left(x\right)\left({\left(\frac{\theta F_{X}^{\theta -1}\left(x\right)f_{X}\left(x\right)}{F_{X}^{\theta }\left(u\right)}\right)}^{\alpha -1}-1\right)I_{\left\{0<X<u\right\}}\right].$$

**Proof.**  From (30) we get

$$f_{Y}\left(x\right)=\theta F_{X}^{\theta -1}\left(x\right)f_{X}\left(x\right).$$

It results:

$$H_{\alpha }^{T}\left(Y_{rt}\left(d\right)\right)=-\frac{1}{\left(\alpha -1\right)F_{Y}\left(u\right)}E_{f_{Y}}\left[\left({\left(\frac{f_{Y}\left(x\right)}{F_{Y}\left(u\right)}\right)}^{\alpha -1}-1\right)I_{\left\{0<X<u\right\}}\right]$$

$$=-\frac{\theta }{\left(\alpha -1\right)F_{X}^{\theta }\left(u\right)}E_{f_{X}}\left[F_{X}^{\theta -1}\left(x\right)\left({\left(\frac{\theta F_{X}^{\theta -1}\left(x\right)f_{X}\left(x\right)}{F_{X}^{\theta }\left(u\right)}\right)}^{\alpha -1}-1\right)I_{\left\{0<X<u\right\}}\right].$$

□

## 5. Applications

We used a real database from [18], representing the Danish fire insurance losses recorded during the 1980–1990 period [44,45,46], where losses are ranged from MDKK 1.0 to 263.250 (millions of Danish Krone). The average loss is MDKK 3.385, while 25% of losses are smaller than MDKK 1.321 and 75% of losses are smaller than MDKK 2.967.

The data from the database [18] were fitted by using a Weibull distribution and the maximum likelihood estimators of the shape $\hat{c}=0.3192$, and scale parameters of the distribution $\hat{\tau }=0.9585$ were obtained.

The results displayed in Table 1, Table 2, Table 3, Table 4 and Table 5 can be used to compare the values of the following entropy measures:

- The Tsallis entropy $H_{\alpha }^{T}\left(X\right)$ corresponding to the random variable *X* which models the loss;
- The Tsallis entropy of the left-truncated loss and, respectively, censored loss random variable corresponding to the per-payment risk model with a deductible *d*, namely $H_{\alpha }^{T}\left(X_{lt}\left(d\right)\right)$ and, respectively, $H_{\alpha }^{T}\left(X_{lc}\left(d\right)\right)$;
- The Tsallis entropy of the right-truncated and, respectively, censored loss random variable corresponding to the per-payment risk model with a policy limit *u*, denoted by $H_{\alpha }^{T}\left(X_{rt}\left(u\right)\right)$ and, respectively, $H_{\alpha }^{T}\left(X_{rc}\left(u\right)\right)$;
- The Tsallis entropy of losses of the right-truncated loss random variable corresponding to the per-loss risk model with a deductible *d* and a policy limit *u*, $H_{\alpha }^{T}\left(X_{lr}\left(d,\;u\right)\right)$.

In the case of the Weibull distribution, for the parameter values λ=0.9585 and γ=0.3192 for d=1.1–1.5,u=10,u=15,u=20,u=25 and for different values of the Tsallis entropy parameter α located in the neighborhood of the point 1, we draw the following conclusions. The values of the Tsallis entropy for α=1 correspond to those obtained in [18]. Moreover, we remark that, for values of the Tsallis parameter α lower than 1, the values of the corresponding entropy measures increase. Moreover, for values of the parameter α greater than 1, the values of the corresponding entropy measures decrease, as we can notice from Figure 3, too. This behavior allows a higher degree of flexibility for modeling the loss-truncated and loss-censored random variables in actuarial models.

## 6. Conclusions

In this paper, an entropy-based approach for risk assessment in the framework of loss models and survival models involving truncated and censored random variables was developed.

By using the Tsallis entropy, the effect of some partial insurance schemes, such as inflation, truncation and censoring from above and truncation and censoring from below was investigated.

Analytical expressions for the per-payment and per-loss entropies of losses were derived. Moreover, closed formulas for the entropy of losses corresponding to the proportional hazard rate model and the proportional reversed hazard rate model were obtained.

The results obtained point out that entropy depends on the deductible and the policy limit, and inflation increases entropy, which means the increase in the uncertainty degree of losses increases compared with the case without inflation. The use of entropy measures allows risk assessment for actuarial models involving truncated and censored random variables.

We used a real database representing the Danish fire insurance losses recorded between 1980 and 1990 [44,45,46], where losses range from MDKK 1.0 to 263.250 (millions of Danish Krone). The average loss is MDKK 3.385, while 25% of losses are smaller than MDKK 1.321 and 75% of losses are smaller than MDKK 2.967.

The data were fitted using the Weibull distribution in order to obtain the maximum likelihood estimators of the shape $\hat{c}=0.3192$ and scale parameters of the distribution $\hat{\tau }=0.9585$.

The values of the Tsallis entropies for α=1 correspond to those from [18], while as the α is lower than 1 the values of the entropies will increase and, as the α is bigger than 1, the values of the entropies will decrease, as we can notice from the Figure 3, too.

The paper extends several results obtained in this field; see, for example, Sachlas and Papaioannou [18].

The study of the results obtained reveals that for parameter values α≠1 the Tsallis entropy corresponding to the right-truncated loss random variable is increasing with respect to the value of the policy limit *u*. On the other side, for α=1, the Tsallis entropy, which reduces to the Shannon entropy measure, is decreasing with respect to *u*. From an actuarial perspective, when the policy limit increases, the risk of the insurance company also increases, therefore the entropy of losses increases, too. The detected behavior proves that the Tsallis entropy approach for evaluating the risk corresponding to the right-truncated loss random variable is more realistic.

Therefore, we can conclude that the Tsallis entropy approach for actuarial models involving truncated and censored random variables provides a new and relevant perspective, since it allows a higher degree of flexibility for the assessment of risk models.

## Figures and Tables

**Table 1:** Tsallis entropy values for the Weibull distribution for: λ=0.9585,γ=0.3192,d=1.1.

α	***u***	$H_{\alpha }^{T}\left(X\right)$	$H_{\alpha }^{T}\left(X_{\mathrm{lt}}\left(d\right)\right)$	$H_{\alpha }^{T}\left(X_{\mathrm{lc}}\left(d\right)\right)$	$H_{\alpha }^{T}\left(X_{\mathrm{rt}}\left(u\right)\right)$	$H_{\alpha }^{T}\left(X_{\mathrm{rc}}\left(u\right)\right)$	$H_{\alpha }^{T}\left(X_{\mathrm{lr}}\left(d,\;u\right)\right)$
	10	5.434	5.5005	5.3837	3.7994	4.1067	4.0564
0.5	15				4.5725	4.7622	4.712
	20				4.9845	5.09140	5.0411
	25				5.2006	5.2582	5.2079
	10	2.5446	2.5778	2.5156	2.2220	2.3504	2.3214
0.9	15				2.4306	2.488	2.4591
	20				2.505432	2.52792	2.4989660524
	25				2.5156	2.5314	2.5396
	10	2.20865	2.2369	1.091	2.316	2.0827	2.0792
1	15				2.2534	2.1767	2.1732
	20				2.22484	2.20041	2.1969
	25				2.2140	2.2064	2.203
	10	1.26474	1.278	1.2521	1.2094	1.24799	1.2353
1.5	15				1.2503	1.2626	1.2499
	20				1.2609475715	1.2644	1.2518
	25				1.2637	1.2647	1.2525
	10	0.8459	0.8524	0.8396	0.8279	0.8433	0.837
2	15				0.8415	0.8457	0.83944
	20				0.8448	0.8459	0.8395
	25				0.8456	0.8459	0.8396

**Table 2:** Tsallis entropy values for the Weibull distribution for: λ=0.9585,γ=0.3192,d=1.2.

α	*u*	$H_{\alpha }^{T}\left(X\right)$	$H_{\alpha }^{T}\left(X_{\mathrm{lt}}\left(d\right)\right)$	$H_{\alpha }^{T}\left(X_{\mathrm{lc}}\left(d\right)\right)$	$H_{\alpha }^{T}\left(X_{\mathrm{rt}}\left(u\right)\right)$	$H_{\alpha }^{T}\left(X_{\mathrm{rc}}\left(u\right)\right)$	$H_{\alpha }^{T}\left(X_{\mathrm{lr}}\left(d,\;u\right)\right)$
	10	5.434	5.5043	5.33	3.7994	4.1067	4.0027
0.5	15				4.5725	4.7622	4.6583
	20				4.98457	5.091	4.9874
	25				5.2006	5.2582	5.1542
	10	2.5446	2.5796	2.4829	2.222	2.35	2.2887
0.9	15				2.43	2.488	2.4264
	20				2.5054	2.5279	2.4662
	25				2.5314	2.5396	2.4779
	10	2.20865	2.2384	1.0621	2.31601	2.0827	2.09548
1	15				2.2534	2.1767	2.1894
	20				2.2248	2.2004	2.2131
	25				2.214	2.2064	2.2192
	10	1.26474	1.2786	1.2364	1.2094	1.2479	1.2197
1.5	15				1.2503	1.2626	1.2343
	20				1.2609	1.2644	1.2362
	25				1.26373	1.2647	1.2364
	10	0.8459	0.85275	0.8311	0.82792	0.8433	0.8285
2	15				0.8415	0.8457	0.8309
	20				0.8448	0.8459	0.8395
	25				0.8448	0.8459	0.8311

**Table 3:** Tsallis entropy values for the Weibull distribution for: λ=0.9585,γ=0.3192,d=1.3.

α	*u*	$H_{\alpha }^{T}\left(X\right)$	$H_{\alpha }^{T}\left(X_{\mathrm{lt}}\left(d\right)\right)$	$H_{\alpha }^{T}\left(X_{\mathrm{lc}}\left(d\right)\right)$	$H_{\alpha }^{T}\left(X_{\mathrm{rt}}\left(u\right)\right)$	$H_{\alpha }^{T}\left(X_{\mathrm{rc}}\left(u\right)\right)$	$H_{\alpha }^{T}\left(X_{\mathrm{lr}}\left(d,\;u\right)\right)$
	10	5.434	5.508	5.2754	3.7994	4.1067	3.9481
0.5	15				4.5725	4.7622	4.6036
	20				4.9845	5.0914	4.9328
	25				5.2006	5.2582	5.0996
	10	2.5446	2.5812	2.4491	2.222	2.3504	2.2549
0.9	15				2.4306	2.488	2.3926
	20				2.5054	2.5279	2.4324
	25				2.5314	2.5396	2.4441
	10	2.2086	2.2398	1.03212	2.316	2.08275	2.11294
1	15				2.2534	2.1767	2.20691
	20				2.2248	2.2004	2.23
	25				2.214	2.2064	2.2366
	10	1.2647	1.2792	1.2199	1.2094	1.2479	1.2031
1.5	15				1.2503	1.2626	1.2178
	20				1.2609	1.2644	1.2196
	25				1.2637	1.2647	1.2199
	10	0.8459	0.853	0.8219	0.8279	0.8433	0.8193
2	15				0.8415	0.8457	0.8218
	20				0.8448	0.8459	0.8219
	25				0.8456	0.8459	0.8219

**Table 4:** Tsallis entropy values for the Weibull distribution for: λ=0.9585,γ=0.3192,d=1.4.

α	*u*	$H_{\alpha }^{T}\left(X\right)$	$H_{\alpha }^{T}\left(X_{\mathrm{lt}}\left(d\right)\right)$	$H_{\alpha }^{T}\left(X_{\mathrm{lc}}\left(d\right)\right)$	$H_{\alpha }^{T}\left(X_{\mathrm{rt}}\left(u\right)\right)$	$H_{\alpha }^{T}\left(X_{\mathrm{rc}}\left(u\right)\right)$	$H_{\alpha }^{T}\left(X_{\mathrm{lr}}\left(d,\;u\right)\right)$
	10	5.43403	5.51157	5.2201	3.79949502	4.106747228	3.892825
0.5	15				4.57256	4.762289	4.54836725
	20				4.98450397	5.0914	4.87748487
	25				5.200639	5.25823	5.04431057
	10	2.54465658	2.5828658	2.414517	2.22206	2.3504043	2.220265
0.9	15				2.430659	2.488092	2.35795
	20				2.50543	2.5279	2.39778476
	25				2.531	2.539648	2.4095
	10	2.20865	2.24113	1.00127	2.31601	2.08275	2.13136
1	15				2.25345	2.17672	2.22534
	20				2.22484	2.20041	2.24902
	25				2.21409	2.20649	2.25511
	10	1.2647449	1.2798459	1.20262623	1.209498976566	1.24799613	1.1858774
1.5	15				1.2626	1.2626	1.2004
	20				1.2609	1.2644668	1.2023481
	25				1.2637	1.2647078	1.2025891
	10	0.8459	0.853296	0.812209	0.82795	0.84332	0.8096
2	15				0.84159	0.8457620	0.8120436
	20				0.84482638	0.84591692	0.81219852
	25				0.84564	0.84592715	0.8122087

**Table 5:** Tsallis entropy values for the Weibull distribution for: λ=0.9585,γ=0.3192 and d=1.5.

α	*u*	$H_{\alpha }^{T}\left(X\right)$	$H_{\alpha }^{T}\left(X_{\mathrm{lt}}\left(d\right)\right)$	$H_{\alpha }^{T}\left(X_{\mathrm{lc}}\left(d\right)\right)$	$H_{\alpha }^{T}\left(X_{\mathrm{rt}}\left(u\right)\right)$	$H_{\alpha }^{T}\left(X_{\mathrm{rc}}\left(u\right)\right)$	$H_{\alpha }^{T}\left(X_{\mathrm{lr}}\left(d,\;u\right)\right)$
	10	5.43403331	5.51497466	5.16428	3.799495	4.1067472	3.8369939886
0.5	15				4.57256	4.762289	4.4925361
	20				4.98450397	5.0914	4.82165
	25				5.2006	5.25823	4.9884794
	10	2.54465658	2.5843899676	2.3792109	2.22206	2.3504	2.1849586855
0.9	15				2.430659	2.488092	2.322646
	20				2.50543	2.52792	2.362478
	25				2.531	2.5396	2.3742
	10	2.20865	2.24240	0.96975	2.31601	2.08275	2.15055
1	15				2.25345	2.17672	2.24452
	20				2.22484	2.20041	2.26821
	25				2.21409	2.20649	2.27429
	10	1.2647449	1.2803868	1.184659	1.209	1.24799613	1.16791
1.5	15				1.25035	1.2626	1.18253
	20				1.2609	1.2644668	1.18438
	25				1.2637	1.2647	1.18462
	10	0.8459	0.85354	0.801878	0.82795	0.8433243	0.79927
2	15				0.8415947	0.845762	0.8017
	20				0.844826	0.8459169	0.801867
	25				0.84564	0.845927	0.801877

## Data Availability

Not applicable.
